# Guiding Through Darkness: How Nurses and Interdisciplinary Teams Foster Healing and Resilience After a Catastrophic Fire

**DOI:** 10.1111/jan.70554

**Published:** 2026-02-18

**Authors:** William H. C. Li, XinYi Xu, Graeme D. Smith

**Affiliations:** ^1^ The Nethersole School of Nursing, Faculty of Medicine The Chinese University of Hong Kong Hong Kong SAR China; ^2^ S. K. Yee School of Health Sciences, Saint Francis University Tseung Kwan O Hong Kong SAR China

Hong Kong is widely regarded as an extremely modern metropolis, renowned for its advanced technological infrastructure and highly developed urban environment. Against this backdrop, the catastrophic fire that erupted at Wang Fuk Court, a public housing complex in Tai Po, on 26 November 2025, appears profoundly incongruous. The blaze engulfed seven of the estate's eight high‐rise residential tower blocks and persisted for 43 h, resulting in 168 fatalities and making nearly 2000 households homeless. This tragedy stands as the worst disaster to have struck Hong Kong in half a century, and the site of the fire now represents a deep wound for the territory and inhabitants.

At the time of the incident, Wang Fuk Court had been undergoing extensive renovation work for over 18 months, and all eight buildings were sheathed in bamboo scaffolding and protective netting, with window openings covered by polystyrene foam boards. Although an independent investigation is currently underway in Hong Kong to ascertain the precise cause of the fire, preliminary findings indicate that the presence of protective netting combined with the use of flammable polystyrene foam boards may have significantly contributed to the rapid spread of the blaze and its intensity. The situation is reminiscent of the Grenfell Tower fire in London in 2017, which resulted from various systemic failures in building design, regulation and maintenance, and its aftermath highlights comparable issues of safety, justice, mental health of survivors, first responders, and others, and long‐term community rebuilding. Although the blaze in Hong Kong has long since been extinguished, the profound trauma it inflicted will take considerably longer to overcome.

Scenes of scorched ruins, grieving residents, and the sea of flowers and paper cranes at Wang Fuk Recreation Ground. These paper cranes serve as potent symbols of hope, commemoration, and resilience within East Asian culture and have been featured in countless news bulletins that continue to bear witness to the tears and farewells of Hong Kong people.

## 1 Invisible Wounds: Psychological Aftermath of the Catastrophe

The fire has left survivors grappling with intense grief and emotional distress, manifested in insomnia, fear, depression, and in some cases, the development of post‐traumatic stress disorder. While the immediate danger has passed and the physical damage at the site can be addressed through reconstruction, the psychological scars left by such a devastating event are far less visible and often more enduring. Following the Grenfell fire, high levels of psychological distress were reportedly prevalent among survivors, especially children and women. If left unattended, these psychological wounds can linger for years, particularly among those who sustained burn injuries and require prolonged recovery and psychological adaptation (Wu et al. 2020).

Despite similarities between the two fires, the initial response of emergency services was dramatically different. The initial reaction in London was widely criticised as dismissive and inadequate. In contrast, Hong Kong witnessed a rapid and coordinated response from its emergency services. The local community in Tai Po was also highly commended for promptly engaging in the initial evacuation, demonstrating high vigilance and community‐mindedness. The Tai Po fire also had a profound impact on frontline emergency service workers, including nurses, leading to extensive mental health strain, highlighting the importance of Hong Kong authorities providing timely and confidential mental health support for these workers.

## 2 When One Suffers, All Respond: Collective Compassion in Action

While the fire inflicted profound pain, it also revealed a deeply moving counterpoint. Hong Kong is often characterised by its relentless pace and atmosphere of detachment. Yet this merciless blaze awakened a reservoir of compassion among its people. Responding to the disaster, the government established the ‘Tai Po Wang Fuk Court Relief Fund’, which within 1 month had received approximately HK$3.8 billion in donations, supplemented by HK$300 million in seed funding from the government, bringing the total to HK$4.1 billion (US$528 million). This fund provides urgent financial assistance to multiple categories of people affected by the disaster.

The aftermath of the fire saw a city united in action. Charitable organisations and major corporations contributed generously, while citizens mobilised with remarkable solidarity, donating an abundance of clothing, food and water to temporary shelters. Meanwhile, restaurants offered free meals, and long queues formed as people volunteered to give blood.

## 3 Temporary Shelters and the Practice of Care

In response to urgent needs, the Hong Kong government opened multiple temporary shelters in Tai Po community halls for affected residents. For several days after the fire, many victims continued to seek assistance. Despite their modest size, these community halls offered a wide range of services: temporary accommodation, a nursing station, a community pharmacy, medical consultations and emotional support. Staff from the Social Welfare Department assisted residents with registration and applications for relief funds as well as relocation to other shelters; the Housing Department facilitated transitional housing applications, and the Immigration Department helped replace lost identity documents. Volunteers distributed meals, and abundantly stocked donations from civic groups were freely available within shelters to those in need, creating a humbling and inspiring scene.

Within these shelters, nursing stations played a pivotal role in ensuring continuity of care. Their responsibilities included monitoring vital signs, documenting physiological and psychological symptoms, and retrieving medical histories and prescriptions via the Electronic Health Record Sharing System. Unsurprisingly, the trauma of the fire led to elevated blood pressure, insomnia and loss of appetite among some survivors. The provision of appropriate nursing care, preventive health education, and psychological support was therefore essential.

## 4 A Cinematic Reflection on Life, Death and Healing

In this context of collective mourning and recovery, cultural expressions have offered meaningful space for reflection. In 2024, a deeply moving film titled *Breaking Through Hell* (also known by the title *The Last Dance*) was released in Hong Kong (Chan 2024). The film's core themes are life, death, and emotional healing. Its title is derived from a traditional Taoist funeral ritual in which the deceased are symbolically guided through nine levels of hell, each representing a distinct moral judgement and form of punishment. In this ritual, Taoist priests perform a series of ceremonial acts to help the soul find peace, such as breaking tiles with a peach wood sword.

Although the film is set in Hong Kong's funeral industry, its deeper story resonates with many viewers, particularly in prompting reflection on fundamental questions of life and death. *Breaking Through Hell* sensitively portrays how individuals confront loss and say farewell. At the same time, it reminds us that healing is not reserved solely for those who are approaching death; the living also need to ‘break through hell’. After the loss of a loved one, those left behind often endure a form of emotional suffering akin to a living purgatory, as they struggle with grief, a sense of emptiness, and the heavy burden of memory.

Against this backdrop of loss and collective compassion, the concept of resilience offers a critical lens through which recovery and healing can be understood. Resilience, conceptualised as a dynamic process, refers to an individual's capacity to adapt to, withstand, and recover from adversity following traumatic events through the mobilisation of personal and social protective resources (Aburn et al. 2016). In the context of the catastrophic fire in Tai Po, strengthening resilience among victims is critical to facilitating psychological recovery and sustaining long‐term mental wellbeing. Hong Kong witnessed widespread evidence of community resilience, representing a collective capacity to withstand, adapt to, and recover in the face of the adversity of the fire, manifested by efforts from NGOs and volunteers. Residents and emergency care professionals spontaneously mobilised to support each other, demonstrating social cohesion, resilience, and resourcefulness. Healthcare professionals, particularly nurses, play a pivotal role in this process by systematically identifying the diverse and evolving needs of individuals experiencing loss. Through timely assessment and care coordination, nurses can facilitate access to appropriate psychosocial and community support, thereby enhancing adaptive coping and resilience throughout the bereavement process (Cheng et al. 2024). A holistic and empowering approach is therefore essential to promote healing across individual, familial, and community levels and to foster the development of responsive and sustainable support systems for this resilient yet vulnerable population.

## 5 How Nurses Realise Compassionate Care in Times of Crisis

In the wake of this catastrophic fire, survivors' journeys through grief underscore the indispensable role of nurses in compassionate care. By offering a compassionate presence, nurses help facilitate emotional repair and healing. Just as the souls of the deceased in the film must be guided through their painful journeys, those who have lost loved ones also require support to heal their internal wounds, find meaning in death and begin life anew.

During the aftermath of the Wang Fuk Court fire, many frontline healthcare professionals voluntarily cancelled their leave to care for the injured, particularly in intensive care units and burn wards, and remained on continuous standby even after scheduled shifts had ended. Their actions exemplify the ethical foundations of nursing—empathy, dignity and unwavering commitment—and illuminate the profession's indispensable role in guiding individuals and communities through life's darkest moments.

## 6 A Flicker of Candlelight

Beyond hospital settings, nurses working in universities and tertiary institutions also responded promptly by volunteering at temporary shelters, offering practical assistance and psychosocial support to those affected by the fire. Although such contributions may appear modest in scale, they reflect a profound solidarity with frontline colleagues and demonstrate the collective strength of the nursing community. These professional acts of service embody the spirit of selfless mutual aid and affirm a shared commitment to alleviating suffering, helping survivors navigate grief, and restoring a sense of normality as swiftly and compassionately as possible.

This collective response resonates deeply with Florence Nightingale's vision of nursing—not merely as an occupation but as a vocation grounded in moral purpose and intimate engagement with human suffering. As Mother Teresa once observed, ‘Not all of us can do great things. But we can do small things with great love’. In the aftermath of the Wang Fuk Court tragedy, this sentiment finds powerful expression in the actions of Hong Kong's nursing community, whose quiet dedication continues to illuminate even the darkest circumstances with a flicker of candlelight.

## Author Contributions

W.H.C.L., X.X. and G.D.S. made substantial contributions to conception and design, or acquisition of data, or analysis and interpretation of data. W.H.C.L., X.X. and G.D.S. involved in drafting the manuscript or revising it critically for important intellectual content. W.H.C.L., X.X. and G.D.S. given final approval of the version to be published. Each author should have participated sufficiently in the work to take public responsibility for appropriate portions of the content. W.H.C.L., X.X. and G.D.S. agreed to be accountable for all aspects of the work in ensuring that questions related to the accuracy or integrity of any part of the work are appropriately investigated and resolved.

## Funding

The authors have nothing to report.

## Conflicts of Interest

The authors declare no conflicts of interest.

## Data Availability

The data that support the findings of this study are available from the corresponding author upon reasonable request.
